
JOURNAL INFORMATION
==============================
Journal ID: Wirtschaftsdienst

Wirtschaftsdienst

EISSN: 1613-978X

ARTICLE INFORMATION
==============================
PMCID: PMC9584275
DOI: 10.1007/s10273-022-3305-2
Article ID: 3305
Article version: 1
Subjects: Ökonomische Trends

Die Entwicklung der Rohstoffpreise seit dem russischen Angriff skrieg auf die Ukraine

The development of commodity prices since the Russian war of aggression on Ukraine

Eurich Marina eurich@hwwi.org

grid.435054.3 0000 0001 2227 8589 Hamburgisches WeltWirtschaftsInstitut (HWWI), Oberhafenstraße 1, 20097 Hamburg, Deutschland
Electronic publication date: 2022 Oct 20
Print publication date: 2022
Volume: 102
Issue: 10
First page: 811
Last page: 812
Copyright: © Der/die Autor:in 2022
License: Open Access: Dieser Artikel wird unter der Creative Commons Namensnennung 4.0 International Lizenz veröffentlicht (https://creativecommons.org/licenses/by/4.0/deed.de).
Open Access wird durch die ZBW — Leibniz-Informationszentrum Wirtschaft gefördert.
License URL: https://creativecommons.org/licenses/by/4.0/

Keywords: O13, Q02
issue-copyright-statement: © The Author(s) 2022

==============================

DOI: 10.1007/s10273-022-3305-2

Wirtschaftsdienst, 2022, 102(10), 811-812
JEL: O13, Q02

Konjunkturschlaglicht

### Die Entwicklung der Rohstoffpreise seit dem russischen Angriffskrieg auf die Ukraine

Vor dem Überfall Russlands auf die Ukraine wurden für das Jahr 2022 für Deutschland noch positive Aufholeffekte der Wirtschaftsleistung durch den Wegfall der Schutzmaßnahmen gegen die globale COVID-19-Pandemie prognostiziert.[1] Inzwischen zeigen sich jedoch die Auswirkungen des Konflikts und der damit verbundenen Sanktionen und Gegensanktionen auf die deutsche Wirtschaft deutlich und lassen somit die Hoffnungen auf positive Aufholeffekte nach der Pandemie sinken.

Besonders stark zeigen sich die Auswirkungen des russischen Angriffskriegs auf den Rohstoffmärkten; hier ist der stärkste Anstieg der Rohstoffpreise seit der Ölpreiskrise 1973 zu verzeichnen. Der Überfall erfolgte zu einem Zeitpunkt, in dem sich die Rohstoffmärkte aufgrund der Auswirkungen der COVID-19-Pandemie und der stockenden Lieferketten in einer ohnehin schon angespannten Lage befanden. Abbildung 1 zeigt die Entwicklung des HWWI Rohstoffpreisindex und der Subindizes für Energie, Industrierohstoffe sowie Nahrungs- und Genussmittel 2022.

Die Preise der Indizes sind mit den durchschnittlichen Importen (2017 bis 2019) aus Drittländern in die Länder der europäischen Währungsunion (EWU) gewichtet, um die Bedeutung der einzelnen Preisänderungen für die EWU-Länder widerzuspiegeln. Mit dem Beginn des Kriegs (grau gestrichelte Linie) ist der Rohstoffpreisindex bis zum 8.3.2022 um 56,8 % angestiegen. Grund hierfür ist vor allem die starke Gewichtung der Energierohstoffe, die einen Importanteil von 74,5 % ausmachen. Dies unterstreicht die hohe Bedeutung Russlands als Energielieferant für die EWU-Länder.

### Energierohstoffe

Ein differenziertes Bild ergibt sich bei Betrachtung der Abbildung 2. Hier ist der Energierohstoffindex in seine Einzelbestandteile Rohöl (Anteil: 56,23 %), Erdgas (Anteil: 14,07 %) und Kohle (Anteil: 4,16 %) aufgegliedert und ab dem Jahr 2020 dargestellt. Die Preise für Energierohstoffe wiesen demnach bereits vor Beginn des Ukrainekriegs eine hohe Volatilität auf und befanden sich aufgrund einer erhöhten Nachfrage bei geringem Angebot bereits vor dem Überfall Russlands auf einem hohen Niveau.

Der starke Preisanstieg (vgl. Abbildung 3) ab dem 24.2.2022 zeigt die Bedeutung Russlands als einer der größten Energielieferanten in diesem Markt. Am 8.3.2022 erreichte der Preis für Erdgas ein lokales Maximum; danach fielen die Preise zunächst wieder, wodurch sich der Subindex kurzzeitig stabilisierte. Dies ändert sich jedoch mit steigenden Unsicherheiten durch reduzierte Liefermengen russischen Erdgases ab Ende Juni bis hin zum

1 HWWI Konjunkturprognose vom 2.3.2022.

© Der/die Autor:in 2022. Open Access: Dieser Artikel wird unter der Creative Commons Namensnennung 4.0 International Lizenz veröffentlicht (creativecommons.org/licenses/by/4.0/deed.de).

Open Access wird durch die ZBW – Leibniz-Informationszentrum Wirtschaft gefördert.

Abbildung 1
**Entwicklung des HWWI-Rohstoffpreisindex**
US-$-Basis, Indexwert 2.1.2020=100

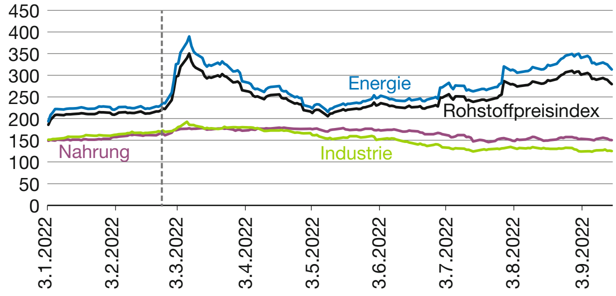

Grau gestrichelte Linie: Tag des Einmarsches Russlands in die Ukraine.

Quelle: Hamburgisches WeltWirtschaftsInstitut (HWWI).

Abbildung 2
**Entwicklung der Energierohstoffe**
US-$-Basis, Indexwert 2.1.2020=100

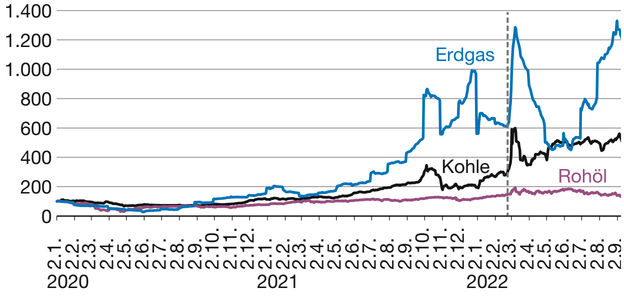

Grau gestrichelte Linie: Tag des Einmarsches Russlands in die Ukraine.

Quelle: Hamburgisches WeltWirtschaftsInstitut (HWWI).

Abbildung 3
**Rohstoffe mit den höchsten durchschnittlichen Preisanstiegen**

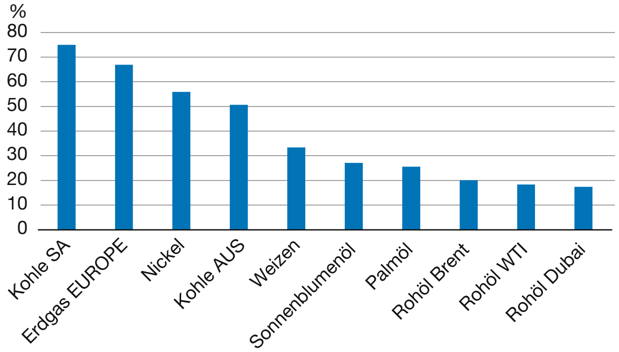

Die Abbildung zeigt die zehn Rohstoffe mit den höchsten Preisanstiegen des durchschnittlichen Preises vor dem Krieg (24.1.2022-23.2.2022) zu dem durchschnittlichen Preis nach dem Krieg (24.2.2022-24.3.2022).

Quelle: Hamburgisches WeltWirtschaftsInstitut (HWWI).

vollständigen Stopp der russischen Erdgaslieferungen Anfang September. Die dadurch entstandene Angebotsverknappung löste einen erneuten starken Anstieg bis fast an den aktuellen Rand aus. Preisdämpfend wirkten sich zuletzt gefüllte Gasspeicher, LNG-Lieferungen und die abnehmende Unsicherheit aus. Die Intention der europäischen Länder, Einsparpotenziale zu nutzen sowie die Diskussion über eine mögliche Deckelung des Erdgaspreises könnten sich ebenfalls preisdämpfend auswirken.

Eine etwas andere Entwicklung ist bei dem Subindex für Kohle zu beobachten. Das Embargo für den Import russischer Kohle sowie eine verstärkte Nachfrage als Ersatz für die Stromerzeugung ließ auch hier die Preise mit am stärksten ansteigen (vgl. Abbildung 3). Jedoch zeigte sich hier keine Erholungsphase, so wie sie für Erdgas zu erkennen ist. Die boomende Nachfrage nach einem risikoarmen Rohstofflieferanten scheint hier zu einem stetigen Preisanstieg beizutragen.

Für die Rohölpreise ergibt sich wiederum ein differenzierteres Bild. Während die Ölpreise zu Beginn des Kriegs unter anderem durch das Ölimportembargo vieler Staaten gegenüber Russland die Preise deutlich steigen ließen, haben sich die Ankündigung der US-Regierung zur Freigabe der strategischen Ölreserven und die zumindest vorübergehende Erhöhung der Ölproduktion der Organisation der erdölexportierenden Länder (OPEC) preisdämpfend ausgewirkt. Vor allem dürfte der Rückgang der Ölpreise aber auch auf die ungünstigeren Konjunkturerwartungen der weltweiten Industrie zurückzuführen sein. Dieser Effekt wird durch die derzeitigen Zinserhöhungen der Zentralbanken im Zuge der Bekämpfung der ungewöhnlich hohen Inflation noch verstärkt.

### Industrierohstoffe

Russland gehört zu den weltweit größten Produzenten von Industriemetallen wie Aluminium und Nickel, die in dem Subindex für Industrierohstoffe enthalten sind. Vor allem die Preise für Nickel, Aluminium und Zink stiegen auf Rekordhöhen (vgl. Abbildung 3). Nachdem der Subindex für Industrierohstoffe am 7.3.2022 seinen Maximalwert erreichte, sank dieser bis zum aktuellen Rand wieder ab. Die weltweit schwachen Konjunkturaussichten lassen die Nachfrage nach Industrierohstoffen weiter sinken, sodass der Subindex am aktuellen Rand bereits unter das Vorkriegsniveau fiel.

### Nahrungs- und Genussmittel

Auch der Index für Nahrungs- und Genussmittel zeigte seit dem Überfall Russlands einen Anstieg, dieser war jedoch weniger stark ausgeprägt als der der übrigen Subindizes. Vor allem die Preise für Weizen, Mais und Öle und Ölsaaten erklären den Anstieg; diese Produkte stammen zu erheblichen Teilen aus Russland und der Ukraine. Auch hier sind die Preise aber im Laufe der Zeit wieder stetig gesunken, wozu vor allem die Wiederaufnahme der Getreideexporte aus der Ukraine beigetragen haben dürfte. Somit hat sich der Index für Nahrungs- und Genussmittel heute ungefähr auf Vorkriegsniveaus eingependelt. Zwischenzeitliche Volatilität entstanden hier durch Ausfuhrstopps einiger Exportländer wie Indonesien, Indien, Ägypten oder Algerien, um mögliche Versorgungsknappheiten innerhalb dieser Länder zu verhindern.

Zusammenfassend lässt sich festhalten, dass sich der Rohstoffindex immer noch auf einem erhöhten Niveau befindet (+28 % Stand: 16.09.2022 im Vergleich zum Tag vor dem Krieg), was aber vor allem auf die immer noch hohen Energiepreise, speziell für Erdgas, zurückzuführen ist. Zwar zeigt sich ein leichter Abwärtstrend. Es ist aber fraglich, ob sich dieser fortführt. Generell ist mit einem eher hohen Niveau der Rohstoffpreise in allen Bereichen zumindest für das nächste Jahr zu rechnen, insbesondere dann, wenn die kriegerischen Auseinandersetzungen länger anhalten.[2] Aber selbst dann, wenn der Krieg zu einem Ende käme, ist kaum damit zu rechnen, dass westliche Staaten schnell wieder zum Einkauf günstiger Rohstoffe aus Russland zurückkehren werden. Ansonsten wird für die Entwicklung der Rohstoffpreise speziell für Energie eine wichtige Rolle spielen, welche Einsparpotenziale genutzt und wie einzelne Energieträger substituiert werden können.

Marina Eurich
Hamburgisches WeltWirtschaftsInstitut (HWWI)

2 World Bank Group (2022), Commodity Markets Outlook, April, https://openknowledge.worldbank.org/bitstream/handle/10986/37223/CMO-April-2022.pdf (23. September 2022).
